# Enterotype-Dependent Probiotic-Mediated Changes in the Male Rat Intestinal Microbiome In Vivo and In Vitro

**DOI:** 10.3390/ijms25084558

**Published:** 2024-04-22

**Authors:** Nikolay Kolzhetsov, Natalia Markelova, Maria Frolova, Olga Alikina, Olga Glazunova, Lubov Safonova, Irina Kalashnikova, Vladimir Yudin, Valentin Makarov, Anton Keskinov, Sergey Yudin, Daria Troshina, Viktoria Rechkina, Viktoria Shcherbakova, Konstantin Shavkunov, Olga Ozoline

**Affiliations:** 1 Laboratory of Functional Genomics of Prokaryotes, Institute of Cell Biophysics of the Russian Academy of Sciences, Federal Research Center “Pushchino Scientific Center for Biological Research of the Russian Academy of Sciences”, 142290 Pushchino, Russia; kolya.kolzhecov@mail.ru (N.K.); markelova.n.y@gmail.com (N.M.); mosmasha@mail.ru (M.F.); alikina.olga@mail.ru (O.A.); glazunova.olga.a@gmail.com (O.G.); shavkunovks@gmail.com (K.S.); 2 Federal State Budgetary Institution “Centre for Strategic Planning and Management of Biomedical Health Risks” of the Federal Medical and Biological Agency, 119121 Moscow, Russia; lsafonova@cspfmba.ru (L.S.); igkalashnikova@cspfmba.ru (I.K.); vyudin@cspfmba.ru (V.Y.); makarov@cspfmba.ru (V.M.); keskinov@cspfmba.ru (A.K.); info@cspfmba.ru (S.Y.); 3 Faculty of Biotechnology, Lomonosov Moscow State University, 119991 Moscow, Russia; darya.troshina02@mail.ru; 4 Laboratory of Anaerobic Microorganisms, Institute of Biochemistry and Physiology of Microorganisms of the Russian Academy of Sciences, Federal Research Center “Pushchino Scientific Center for Biological Research of the Russian Academy of Sciences”, 142290 Pushchino, Russia; vicyle4ka@gmail.com (V.R.); vshakola@gmail.com (V.S.)

**Keywords:** male rat intestinal microbiota, enterotypes, probiotics, *Lacticaseibacillus*, *Bifidobacterium*, enterotype shift, artificial microbiomes

## Abstract

Beneficial properties of lactic acid bacteria have been known long ago, but particular interest in probiotics has arisen in the last two decades due to the understanding of the important role of intestinal microflora in human life. Thus, the ability of probiotics to support healthy homeostasis of gut microbiomes has received particular attention. Here, we evaluated the effect of a probiotic consisting of *Bifidobacterium longum* and *Lacticaseibacillus paracasei* on the gut microbiome of male rats, assessed their persistence in the fecal biota, and compared probiotic-mediated changes in vitro and in vivo. As expected, microbiomes of two enterotypes were identified in the feces of 21 animals, and it turned out that even a single dose of the probiotic altered the microbial composition. Upon repeated administration, the E1 biota temporarily acquired properties of the E2 type. Being highly sensitive to the intervention of probiotic bacteria at the phylum and genus levels, the fecal microbiomes retained the identity of their enterotypes when transferred to a medium optimized for gut bacteria. For the E2 biota, even similarities between probiotic-mediated reactions in vitro and in vivo were detected. Therefore, fecal-derived microbial communities are proposed as model consortia to optimize the response of resident bacteria to various agents.

## 1. Introduction

The physiological role of the gut microbiome, which has gained special attention in the last decades, can hardly be overestimated. Numerous bacteria, archaea, and fungi living in the intestine affect the functioning of the host body via the secretion of an enormous spectrum of metabolites [1,2] and are important to its welfare. Various bacterial species produce vitamins, organic acids, and other beneficial metabolites [3], take part in the utilization of diverse compounds (lactate, cellulose, glycans, etc.), maintain or improve the immunity status [4,5], compete with potential pathogens and even regulate the expression of various host’s genes [6,7,8]. Some studies have testified that the role of the microbiome is even more significant than human genetics in explaining metabolic variations between individuals [9].

Although intestinal communities are fairly stable, their balanced composition can be disrupted in response to antibiotic use or dietary changes [10]. This can lead to dysbiosis due to the expansion of pathogens [10,11,12,13] or cause a range of other disorders and diseases [14,15]. It is already known that unbalanced bacterial composition can be “corrected” using fecal biota transplantation [16,17,18,19,20,21]. This is promising but requires the development of effective methods for preserving the biodiversity of healthy microbiomes and culturing them ex vivo before transplantation. While often less efficient, classical methods of therapy based on oral administration of probiotics are still the most widely used [22,23,24,25].

Probiotic bacteria, often assuming a combination of representatives of *Lactobacillus*/*Lacticaseibacillus* and *Bifidobacterium* genera [26,27], can compete with pathogens and even kill them by bacteriocides [28], induce the immune system, produce metabolites helpful for other members of the host’s community, etc. [29,30]. It is likely that “good” bacteria not only improve the composition of the intestinal microflora [31,32] but also stimulate the stress resistance of the host [33], assist in anti-tumor therapy [25], prevent the development of pre-cancer conditions, and even improve cognitive abilities [34]. The latter corresponds to the idea of the existence of a bidirectional microbiota-gut-brain axis [35]. In animal models, anti-stress effects were demonstrated by *Bifidobacterium infantis* [36], *B. longum* [33], *B. breve* [37], *Lacticaseibacillus rhamnosus* [38,39], *L. paracasei* [39] and other related species. A mixture of *Lactobacillus helveticus* with *B. longum* reduced anxious behavior in response to physical stressors in rats and depressive symptoms in humans [40], while the uptake of *L. casei* for two months reduced exam stress in students [41]. However, there is also evidence that probiotics have no effect on stress resistance in healthy young adults [42,43]. This may be due to the inability of the probiotic bacteria to integrate into the resident microflora or the inability of the individual microbiome to perceive symbiotic interactions with probiotic strains.

Assessment of variations in the microbial communities of the human intestine has led Arumugam and co-authors [44] to a suggestion that they can be divided into three distinct groups named “enterotypes”. Each of them could be characterized by the dominant genera of bacteria present, with the prevalence of *Bacteroides*, *Prevotella,* or *Ruminococcus*. A less straightforward concept has also been proposed [45], according to which the microbial entity is non-uniform in density and possesses a non-discrete structure. Apart from that, enterotypes of various human populations have been shown to depend on genetics [46], dietary preferences [47], climate, environment, and other factors [48]. It is to be expected that different enterotypes will respond differently to the introduction of particular probiotics [49] due to the features of their microbial composition and the resulting difference in interspecific communications. A number of studies have provided some evidence for this. For example, in response to the consumption of low-fat yogurt containing lactic acid bacteria with subcomponents including *Streptococcus thermophilus* and *L. bulgaricus*, the content of *Faecalibacterium*, *Eggerthella* and *Leuconostoc* increased in the human intestines of enterotype 1, while representatives of *Streptococcus*, *Agathobacter* and *Christensenella* correlated with the presence of *Prevotella* dominant in the second enterotype [50]. Apparently, this means that therapeutic probiotic compositions must be developed, taking into account the patients’ enterotypes.

Attempts to estimate the stability of human enterotypes undertaken so far have provided contradictory results in some studies. On the one hand, most bacterial strains are known to persist in the gut biota for many years or decades [51] and 5-day dietary administration of bifidobacteria and lactobacilli did not lead to the expansion of these probiotics [52]. Another work testified that even a 6-month implementation of the Nordic diet did not cause significant changes in the abundance of enterotype-dominant taxa [53].

At the same time, a study in patients with large artery atherosclerotic stroke registered a shift from enterotype 2 (*Prevotella*) to enterotype 3 (*Ruminococcus*) [54], which means the ability of the microbiota to switch established interspecies regulatory networks to adapt to the physiology state of the host.

The feature of differentiation is not limited to human intestinal communities [55]. For instance, two enterotypes have been described in goats [56], showing a correlation with animal growth rate and a number of biochemical parameters, including metabolism of sugars and biosynthesis of amino acids. There are several studies characterizing rat intestinal microbiome stability under different conditions or pathological states [57,58,59]. Of direct relevance to our study are the data obtained by Choi and co-authors [60], who identified two enterotypes in the intestinal microbiota of 24 male rats, whereas a similar number (25) of female animals were clustered into three groups. Fan and co-authors [61] identified three enterotypes in the intestine of *Ochotona curzoniae* and showed that the transition of animals to laboratory nutrition altered the composition of dominant bacteria, resulting in a “single enterotype”. However, adding a natural diet component (swainsonine) to the nutrition partially restored the original homeostasis.

All in all, it is commonly believed that gut microbiomes tend to restore their balanced state, which is more or less person-specific and dependent on a huge variety of factors [62]. A number of studies have demonstrated individual variability in response to diets, pathologies, and other interventions, which is due to the unique characteristics of human microbiomes [63,64]. As a result, the short-term and especially long-term effects of probiotics are very difficult to predict, and this is one of the main reasons why, despite the multiple pieces of evidence of the benefits from probiotics, the dynamics of their progression is not quite clear.

Every hard task is to be worked out via consequent smaller steps. Hence, the creation of simplified artificial microbiomes has proven to be efficient in elucidating the roles of particular species in the natural microbiomes [65,66] and identifying taxa that specifically respond to therapeutic treatments. One such study allowed balancing as many as 104 most common gut-colonizing species, including 5 *Bifidobacterium* and 1 *Lactobacillus* species, together into a sustainable community [67] which, upon challenging with a human fecal sample, increased as a stable consortium of up to 119 bacteria with enhanced stability to external fecal communities and resistance against pathogenic *Escherichia coli*. The main limitation of this approach is the absence of individuality.

Therefore, the main objective of our study was to compare the probiotic effects in vivo and in vitro, using natural fecal samples from probiotic-treated rats and biota obtained from each sample transferred to laboratory media and treated with probiotic ex vivo. Although rats, well-studied model organisms, have been extensively used for assessment of the various effects from the introduction of beneficial bacteria [68,69,70], there are still many questions to address. To accomplish the main task, it was necessary to assess the sensitivity of the intestinal microbiota to probiotic bacteria in an enterotype- and dose-dependent manner, as well as to formalize the information obtained for subsequent comparison. Upon this analysis, we noticed that the introduction of *B. longum* as part of probiotic consortia decreased, rather than increased, the percentage of bifidobacteria in the E2 microbiota and registered reversible probiotic-mediated shift of the E1 bacterial communities towards E2. These observations, along with observed preservation of enterotype identity in vitro and similarity in the E2 response in vivo and in vitro, are the main contributions to the field from this study.

## 2. Results

### 2.1. Rat Intestinal Microbiomes of Two Enterotypes Responded Differently to Probiotic Administration In Vivo

**Figure 1:**
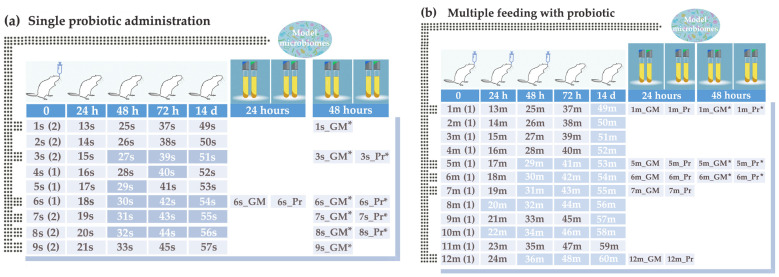
Sample collection protocol and sample names. A single (**a**) or the first (**b**) administration of the probiotic bacteria was carried out at point 0, when control samples were collected (1s–9s and 1m–12m, respectively). Enterotypes defined as described below are indicated in parentheses. Samples 13s–57s in (**a**) were taken at the indicated time points without additional probiotic administration. Samples 13m–60m in (**b**) were collected at the same time but in parallel with the introduction of two additional doses of probiotics, as indicated in the top row. Samples printed in black (light background) were used for analysis in this study. “Model microbiomes”: samples transferred to Gut Microbiota Medium and cultured for 24 (GM) or 48 h (GM*) without the probiotic, and samples cultured with the probiotic for 24 (Pr) or 48 (Pr*) hours.

Fecal samples from 21 healthy male rats were individually collected before intragastric administration of the probiotic, composed of new isolates of bacteria belonging to *L. paracasei* and *B. longum* species. After 24 h of treatment, the first experimental samples were collected. The rats were then randomly divided into two groups (Figure 1). Nine animals of the first group (Figure 1a) received only one dose of the probiotic and were allowed to rebuild their microbiomes over two weeks. The rats were kept under conditions similar to the other animals and were given the same diet without any treatment. Their fecal samples were individually collected 24, 48, and 72 h after treatment, as well as after two weeks of recovery. Twelve rats of the second group received three doses of the probiotic at 24-h intervals and were also kept until two weeks without additional treatment (Figure 1b). As a result, we collected 110 fecal samples, which were frozen (−40 °C) and individually stored until use.

**Figure 2:**
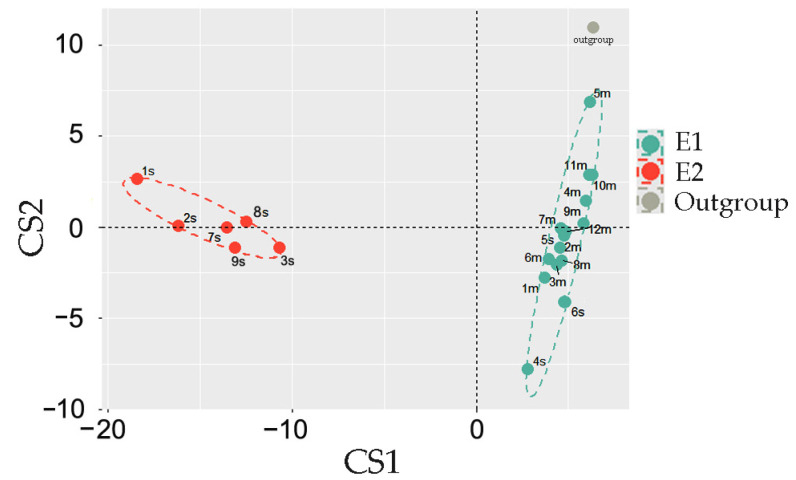
Enterotypes of 21 control microbiomes (samples 1s–9s and 1m–12m in Figure 1). Sample 5m with deliberately changed percentages of two genera was used as an outgroup.

When the feeding experiment was finished, metagenomic DNA was isolated from control samples and used for 16S rRNA taxonomic analysis. Amplicons were classified with EPI2ME v. 3.6.2 software using the Fastq WIMP algorithm. The Jensen-Shannon distances between samples were then calculated based on different in size and composition sets of potential marker taxa. The Calinski–Harabasz indices computed at this stage revealed variability in the optimal number of potential clusters, but using 208 genera with an abundance of ≥0.1% in at least one dataset provided the optimum with the two enterotypes (Figure 2), consistent with the two enterotypes identified in the gut microbiomes of male rats [60].

**Figure 3:**
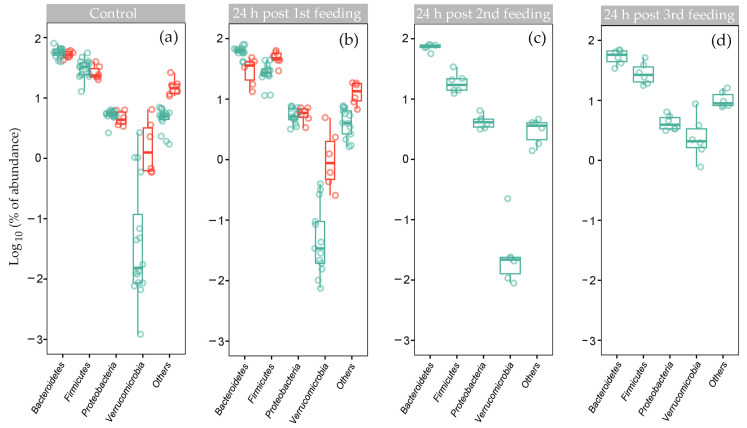
The main phyla of the fecal microbiomes belonging to two enterotypes differentially responded to probiotic administration. (**a**,**b**) The abundance of indicated phyla (**a**) in the E1 (cyan) and E2 (red) fecal biota (samples 1s–9s and 1m–12m) and their responses (**b**) to the single dose of probiotic (samples 16s, 17s, 21s, 13m–19m, 21m, 23m, 24m and 13s–15s, 19s, 20s, 21s for E1 and E2 microbiomes, respectively). (**c**,**d**) The abundance of the dominant phyla 24 h after the second (**c**) and third (**d**) probiotic administration estimated for E1 microbiomes (samples 25m–28m, 33m, 35m and 37m–40m, 45m, 47m, respectively). Statistical data are presented in Supplementary Table S1.

The majority of microbiomes in our set belonged to the enterotype with a low abundance of *Verrucomicrobia*, characteristic of Enterotype 1 (E1) in [60]. Therefore, we retained the same name for the main cluster, although we did not observe a clear dominance of *Firmicutes* over *Bacteroidetes* (Figure 3a, Supplementary Table S1), as previously reported [60]. Twelve rats with E1 microbiomes received three doses of the probiotic (Figure 3b–d), and samples from rats 4s and 5s were available to trace their recovery from a single probiotic administration. Unfortunately, the group with the second enterotype (E2) accidentally included only rats that received a single dose of the probiotic. By excluding the possibility of tracing the dose-dependent response of their microbiomes, this made it possible to trace the dynamic of the E2 biota recovery after a single probiotic treatment.

Even a single dose of the probiotic changed the ratio of *Bacteroidetes* to *Firmicutes* (B/F) in the E2 microbiomes from 2.1 to 0.77 (*p* = 6.76 × 10^−7^) without a significant effect on the presence of *Proteobacteria* and *Verrucomicrobia* (Figure 3a,b). However, in the E1 biota, *Verrucomicrobia* decreased their divergence, which was further reduced after the second feeding (Figure 3c), when the B/F ratio significantly (*p* = 8.4 × 10^−4^) increased from 2.06 to 4.32 (Figure 3c). At the next step, the most dramatic alterations were again observed for *Verrucomicrobia*, whose percentage in the E1 biota increased from 0.05 ± 0.03% to 3.18 ± 1.2% (Figure 3d). Thus, it became clear that the adaptive response of microbiota to *B. longum* and *L. paracasei* is manifested at the phylum level. At the next stage, we evaluated the sensitivity of the gut biota to the introduction of probiotic bacteria at the genus level.

**Figure 4:**
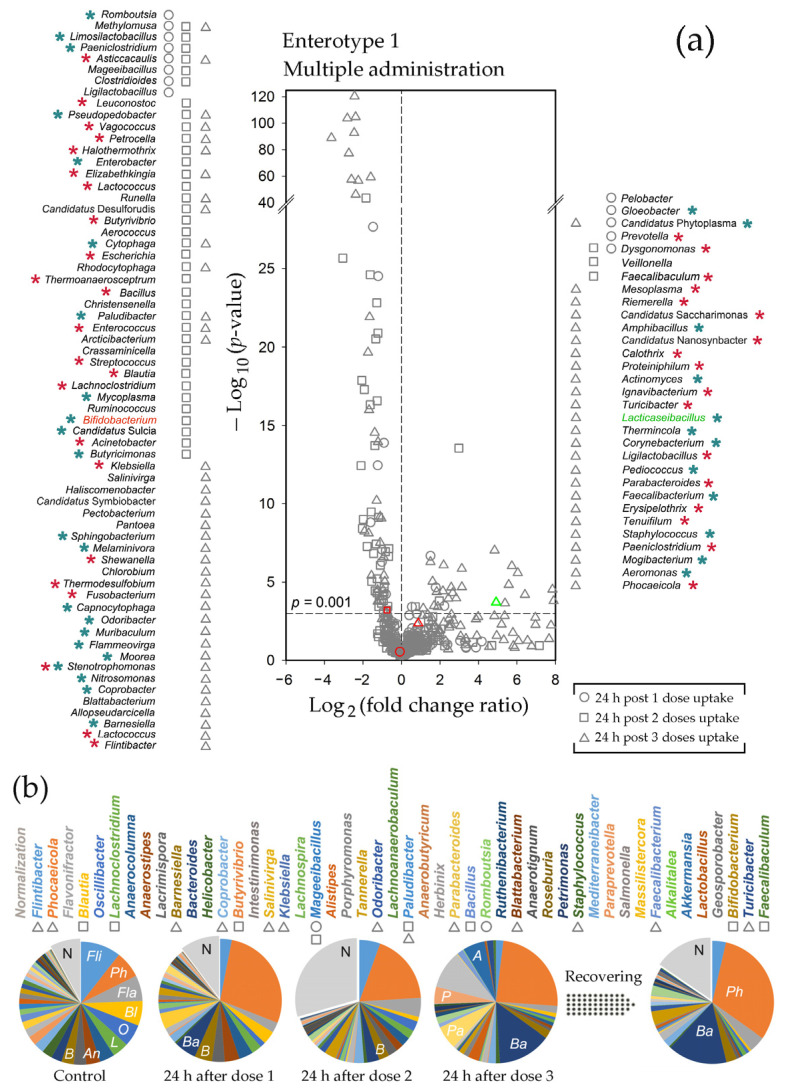
Probiotic-mediated dose-dependent changes in the E1 biota. (**a**) The response of the fecal biota to the introduction of probiotics was determined from the analysis of 13, 6, and 6 paired samples obtained after treatment with 1, 2, and 3 doses of bacteria, respectively. Red and green symbols mark points corresponding to bifidobacteria and lactobacilli. The genera suppressed (left) and stimulated (right) with *p* ≤ 0.001 are listed in descending order of probiotic effect at different stages of feeding. Asterisks indicate the genera that showed a similar (cyan) or an opposite (red) response in the E2 microbiomes (*p* < 0.05). (**b**) Example of dynamic changes in the percentage of 48 genera selected as described in Section 4, plotted in descending order of their percentage in the control sample (11m) and colored identically to the circle diagrams (samples 11m, 23m, 35m, 47m, and 59m). Taxa indicated in the volcano plot (*p* ≤ 0.001) are marked by corresponding symbols. A normalizing sector N was added to the plots to allow direct comparisons of taxa abundances at different time points.

By monitoring 208 genera in the E1 microbiomes, we obtained data from 186 paired samples with reliable numbers of reads and observed a strong and reproducible reaction of bacteria on probiotic administration (Figure 4a). It was dose-dependent, such that only a few taxa were significantly (*p* < 0.001) suppressed or stimulated after the first probiotic administration (circles in Figure 4a), but the second and the third doses (rectangle and triangle symbols, respectively) increased their number to 63 and 31, respectively.

**Figure 5:**
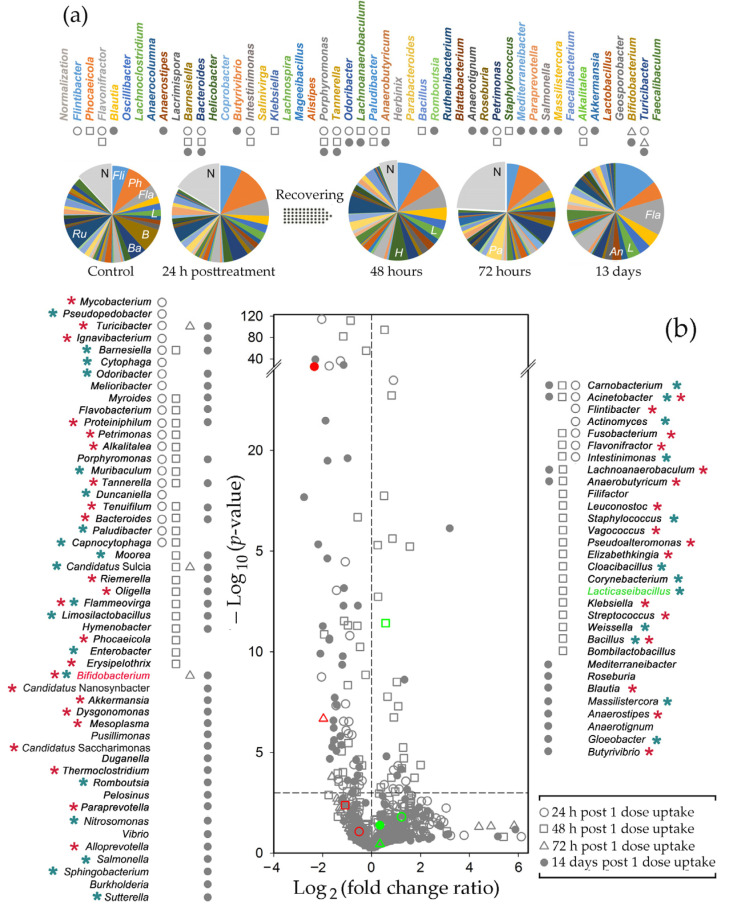
Even a single probiotic treatment altered the composition of the E2 microbiomes, and the changes were retained over 14 days post-treatment. (**a**) Example of dynamic alterations in the percentage of 48 genera (samples 1s, 13s, 25s, 37s and 49s). (**b**) Responses of the E2 biota to single probiotic intervention were assessed using paired samples 1s–13s, 2s–14s, 3s–15s, 7s–19s, 8s–20s and 9s–21s (open circles). Pairs of sample 1s with samples 25s, 37s, and 49s; sample 2s with samples 26s, 38s, and 50s; as well as sample 9s with 33s, 45s, and 57s were used to monitor recovery from probiotic treatment at different time points. See Figure 4 legend for more details.

Figure 4b and Figure 5a show examples of individual microbiome plasticity for 48 genera. Of the six dominant bacteria in the E1 type sample 11m, only *Phocaeicola* (*Ph*) was significantly stimulated by the probiotic. *Flintibacter*, *Blautia,* and *Lachnoclostridium* were highly suppressed at least at one time point (Figure 4a), while *Flavonifractor* (*Fl*) and *Oscillibacter* (*O*) were inhibited. *Bacteroides* (*Ba*), *Paraprevotella* (*P*), and *Parabacteroides* (*Pa*) also showed a reproducible reaction to the probiotic. Their presence in the fecal biota increased already after the first dose, and after the third treatment, they exceeded the control level by 5–7 times (Figure 4b). However, in most cases, the first reaction occurred after the second or third feeding, and not always it was unidirectional. *Akkermansia*, for instance, was inhibited after the first two feedings in 7 of 19 samples, including 23m and 35m microbiomes, but after the third dose, its presence increased in all samples with *p* = 0.03. Belonging to *Verrucomicrobia*, *Akkermansia* contributes to the expansion of this phylum in Figure 3d. Over 11 days of recovery, the percentage of *Akkermansia* reduced to the control level, which was not observed for many other taxa receiving three (Figure 4b) or only one dose of probiotic (Figure 5a).

The total bacterial percentage of 48 representative taxa In control sample 1s with the E2 biota (Figure 5a) was nearly the same as in sample 11m (36.6 and 34.6%, respectively), but their profiles and responses to probiotic were different. In both samples, the first three genera were highly presented, but *Flintibacter* and *Flavonifractor* tended to increase rather than decrease their abundance in response to probiotics, while *Barnesiella* (*B*) and *Ruthenibacter* (*R*) were suppressed rather than stimulated. Such an opposite reaction was observed for many other taxa, including *Anaerostipes* (*An*), which progressively decreased after the first two doses of the probiotic in sample 11m (Figure 4b) but doubled in sample 1s by the end of the experiment (Figure 5a). Some other genera, like *Parabacteroides* (*Pa*), which similarly grew after the first probiotic intervention, demonstrated opposite dynamics later, without any additional intervention in 1s fecal biota. *Helicobacter* (*H*) provided another example of this type. Being strongly inhibited in sample 11m by the first dose, it initially increased its presence by 2.9 times in 1s. Without additional treatment it was suppressed on the third day at the end was at a level 5 times lower than the control. Thus, it is likely that the effect of the probiotic may be delayed and long-lasting.

Statistical processing of data obtained for the E2 microbiomes was based on the analysis of 182 taxa. In this set, more genera showed a negative reaction to the probiotic than in the case of the E1 biota (open circles in Figure 4a and Figure 5b). The next day (rectangles), most remained suppressed, and even 14 days after treatment, many inhibited taxa showed reduced abundance, as observed for *Barnesiella* and *Ruthenibacterium* in Figure 5a. The number of the E2 genera positively responding to the probiotic bacteria was almost the same as in the E1 biota, but their reaction was weaker (FCR typically less than 2), although more uniform across the samples. As a result, two days after probiotic application, when the positive response of the E1 biota was minimal, there was a significant stimulation of many genera, including *Flavonifractor* and *Lachnoclostridium* (Figure 5a,b). The next day, no taxa increased their presence with *p* ≤ 0.001 in the E2 feces, and only three genera were suppressed (Figure 5b). This could be considered as an expected return to the control state. However, 11 days later, the percentage of many taxa with a negative (*Barnesiella*) or positive (*Lachnoclostridium*) reaction to the probiotic changed again (filled circles in Figure 5b). The dynamics of the adaptive response of microbiomes to even a single probiotic intervention may, therefore, be nonlinear over time.

### 2.2. Shaping Fecal Microbiomes, Probiotic Bacteria Were Counteracted by the Resident Biota

**Figure 6:**
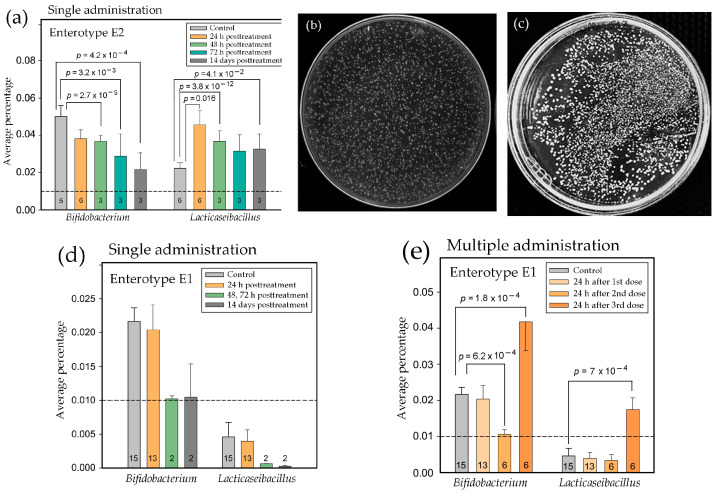
Probiotics promote adaptive changes in the abundance of resident bifidobacteria and lactobacilli. (**a**,**d**,**e**) Dynamics of changes in the percentage of bifidobacteria and lactobacilli in response to single (**a**,**d**) or multiple (**e**) introduction of *B. longum* and *L. paracasei*. Sample numbers are indicated on the bars. Numerical data are presented in Supplementary Table S2. (**b**,**c**) *B. longum* and *L. paracasei* grown anaerobically on TOS-MUP (**b**) or MRS (**c**) media one month after lyophilization.

One of the most surprising results of this study was a transient (Figure 4a) or even gradual (Figure 5b) decrease, rather than the expected expansion, of bifidobacteria in the experimental samples. In the E2 biota, bifidobacteria were among the most inhibited taxa, and Figure 6a shows the opposite effects exerted by a single dose of the drug on bacteria of two probiotic genera.

The percentage of lactobacilli increased as expected on the next day after the probiotic administration, later slightly decreased, but still remained at a level above the control after 11 days without additional portions of probiotic bacteria (Figure 6a). Bifidobacteria, on the contrary, were gradually suppressed from the first day until the end of the experiment. Since month-long storage of the probiotic maintained approximately the same titer of both probiotic bacteria in the co-lyophilized culture (Figure 6b,c), this difference cannot be due to the absence of alive *B. longum* cells in the added bacterial mixture. The dynamics of changes in the abundance of probiotic genera in the E1 microbiomes were different. The background presence of bifidobacteria was lower and remained at the control level on the next day after a single dose (Figure 6d). However, in samples collected at 48 h (28s), 72 h (41s), and 11 days (52s, 53s) posttreatment, their presence decreased (Figure 6d). Together with a decrease in the content of bifidobacteria in samples 25m–28m, 33m, and 35m collected 24 h after the second treatment (Figure 6e), this indicates a transient suppression of this genus by resident microflora, which was excessively compensated after taking the third dose of probiotic (Figure 6e). The presence of lactobacilli in the E1 biota was below the threshold level and tended to decrease in response to the first and second doses of the drug. However, the third administration also provided an expected expansion. Thus, it is likely that the unexpected suppression of bifidobacteria in response to probiotic administration is only part of the pathway for the integration of exogenous bacteria in the resident microbial community.

### 2.3. Multiple Probiotic Administration Can Promote Enterotype Shifts in Resident Microbiomes

**Figure 7:**
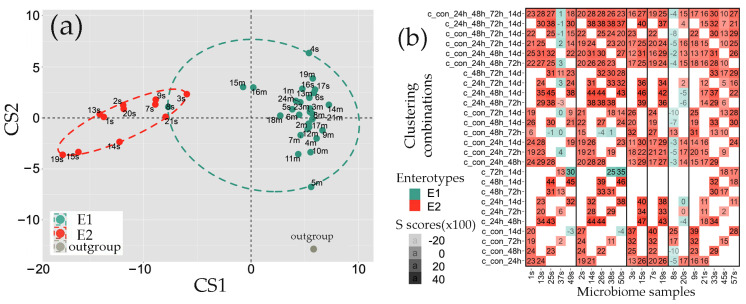
The combined clustering of samples into two groups obtained at different time points of the feeding experiment revealed some instability of the E2 biota. (**a**) Clusters of 21 microbiomes obtained from fecal samples prior to probiotic treatment (set “con”, samples 1s–9s and 1m–12m) and 19 microbiomes of rats that received one dose of probiotic (set “24 h”, samples 13s–17s, 19s, the 20s, 21s, 13m–19m, 21m, 23m, and 24m). (**b**) Heat map showing the silhouette scores (S × 100) for each sample (indicated below) of the original E2 biota. The combination “con_24h” was used for clustering in panel (**a**). Cell color corresponds to the enterotype, for which the target sample had a higher S. White cells correspond to combinations of the sets in which the target sample was absent.

The reverse reaction of bifidobacteria and lactobacilli in the E1 microbiomes after the third dose of probiotic (Figure 6e), together with an increase in the abundance of *Verrucomicrobia* to a level typical for E2 biota at the same time point (Figure 3d), indicated large rearrangements in the bacterial consortium. To assess the extent of these alterations, we performed a combined clustering of the control samples with microbiomes received at different time points. It turned out that automatic clustering of the control samples with the biota of rats fed by only one dose of probiotic already revealed an overlap between the two groups, in which sample 8s, previously defined as type E2 (Figure 2), was assigned to the E1 group (Figure 7a). This meant that its silhouette score (S) in the pooled set (con_24h) was higher in the combined E1 group than in the E2 enterotype (Figure 7a).

This dependence of clustering on the composition of the analyzed sets prompted us to test the stability of enterotype identification for all the samples using all possible combinations of sets obtained at different time points (Figure 7b). Microbiome 8s did show a dependence on the combination of samples used, but all S values justifying its better cohesion with the E1 cluster were negative. Therefore, its assignment to E1 in the con_24h pooled set (Figure 7a) is questionable; moreover, probiotic administration (sample 20s) increased the similarity of this microbiome with the E2 biota (Figure 7b). All the other E2 samples (1s–3s, 7s, and 9s) retained their belonging to the E2 biota the next day after a single dose of the probiotic (Figure 7b). However, on the third day, the sample 37s also showed some tendency towards E1 homeostasis (mostly realized in the combination con_48h_72h), while samples 38s, 49s, and 50s were grouped separately from samples 37s, 45s, and 57s in the set 48h_72h. Since the changed B/F ratio the next day after the single dose (Figure 3a,b) did not change the enterotype of 13s–15s, 19s, and 21s, it is likely that rearrangement at the level of genera (Figure 5b) caused the enterotype shift in samples 38s, 49s, and 50s.

**Figure 8:**
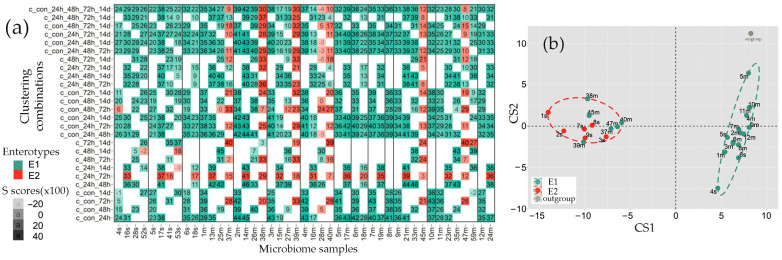
Three doses of probiotics caused a shift in the enterotype of the E1 biota. (**a**) Heat map showing the silhouette scores (S × 100) for each sample (indicated below). The sets obtained at different time points were pooled in combinations indicated on the left. Cell color corresponds to the enterotype, for which the target sample had a higher S. (**b**) Clusters of 21 microbiomes obtained from fecal samples prior to probiotic administration (samples 1s–9s, 1m–12m) and six microbiomes of rats that received three doses of probiotic (samples 37m–40m, 45m, 47m). Cyan dots in the E2 cluster mark samples with changed enterotypes.

In the E1 biota, practically all samples retained belonging to the original type after the single (4s, 5s, and 9s) or the first (13m–19m, 21m, 23m, 24m) doses of probiotic. The next day, two microbiomes (25m and 28m) exhibited some similarity to the E2 biota. However, after the third dose, all the samples showed a collective shift towards the E2 type (Figure 8a,b). Surprisingly, at this time point, even samples 52s and 53s resulting from a single probiotic intake exhibited some clustering instability (Figure 8a).

According to this analysis, the only E1 microbiome analyzed after 11 days of recovery (sample 59m) almost returned to the enterotype of the original sample 11m (Figure 8a), although the corresponding pie chart clearly shows multiple differences in the distribution profile of the 48 genera selected for visualization (Figure 4b). Thus, it became clear that exogenous *B. longum* and *L. paracasei* can transiently alter the enterotypes of the rat intestinal microbiota, but to assess the scale of such changes, an integral approach based on a sufficiently large number of marker taxa is required.

### 2.4. Fecal Microbiomes of Two Enterotypes Transferred to GMM Responded Differently to the Introduction of Probiotic In Vitro

The aim of the last part of the study was to assess the feasibility of using simplified microbiomes extracted from feces as model communities for test systems. Six E1 microbiomes and five bacterial communities belonging to the E2 type were transferred to the medium adapted to gut microbiomes (GMM), and their responses to the intervention of probiotic bacteria in vitro were elucidated. As expected, the environmental change significantly reduced the number of detected genera and the biodiversity in all samples. The number of identified taxa in the E1 and E2 fecal samples decreased to approximately the same values in samples cultured ex vivo for 24 or 48 h (Supplementary Figure S1a). However, Shannon diversity indices (H′) in the E1 microbiomes grown 24 h in GMM decreased from 5.23 ± 0.17 to 3.20 ± 0.24 and increased to 3.78 ± 0.28 after 48 h cultivation (Supplementary Figure S1b). In the E2 samples, after 48 h growth in GMM, H′ decreased from 5.08 ± 0.23 to 2.70 ± 0.17 (Supplementary Figure S1c), providing a statistically significant difference (*p* = 0.01) between the reactions of two enterotypes.

Curiously, in fecal samples, such a difference was also observed in response to probiotic bacteria. Thus, the 2-nd dose of the probiotic caused a temporary decrease in the biodiversity of the E1 microbiomes, which was accompanied by a statistically significant recovery from H′ = 4.28 ± 0.24 to 4.95 ± 0.26 after the third dose (Supplementary Figure S1d). However, a single administration of the probiotic to rats with the E2 biota caused an average increase in diversity to a value of 5.74 ± 0.29 the next day and remained at a slightly elevated level up to 13 days of recovery (Supplementary Figure S1e). The addition of the probiotic in vitro did not affect the biodiversity of ex vivo cultured bacteria (Supplementary Figure S1b,c).

**Figure 9:**
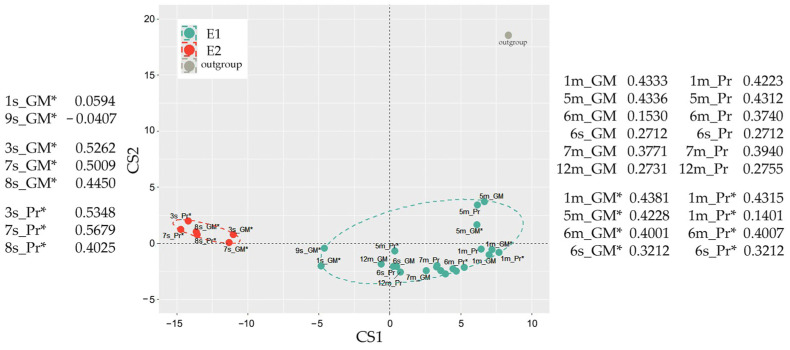
Model bacterial communities transferred into GMM retain their non-overlapping clustering. Samples used for analysis are listed in the plot together with silhouette scores, reflecting their cohesion in the suggested groups.

Most surprising was the fact that the samples had almost the same distribution of ex vivo cultured samples between the two enterotypes (Figure 9) as that of the fecal microbiota (Figure 2). Thus, all the microbiomes recovered from the E1 biota clustered together. Although two samples originated from 1s and 9s fecal biota belonging to the E2 type (1s_GM* and 9s_GM*), shifted into the E1 group, on the principal component analysis plot, they occupy an intermediate position, and 9s_GM has a negative S in the E1 cluster (Figure 9).

A total of 168 (E1) and 173 (E2) genera met the selection criteria for statistical evaluation of the probiotic effect in vitro. Most genera were suppressed in both types of microbiomes (Supplementary Figures S2 and S3). If the cut-off level for the *p*-value was set at 0.05, then the portion of the inhibited taxa reached 58.9% in the revived E1 microbiomes. However, 19.0% of genera exhibited statistically significant expansion, which, like in Figure 4a, was highly variable for additional 8.3% of the stimulated bacteria, including *Lacticaseibacillus* (Supplementary Figure S2). For the E2 microbiomes, these values were 72.8% and 11.0%, respectively (Supplementary Figure S3), indicating that the E2 microbiota may be more sensitive to environmental change or freezing during sample storage. Most of the suppressed bacteria were significantly inhibited in the microbiomes of both enterotypes (cyan asterisks in Supplementary Figures S2 and S3), but there were only five common genera among the bacteria activated in GMM. Thus, it is likely that the specific reaction of the enterotype was realized primarily via expansion rather than survival, and there were genera with the opposite response to changes in the environment (red asterisks in Supplementary Figures S2 and S3).

**Figure 10:**
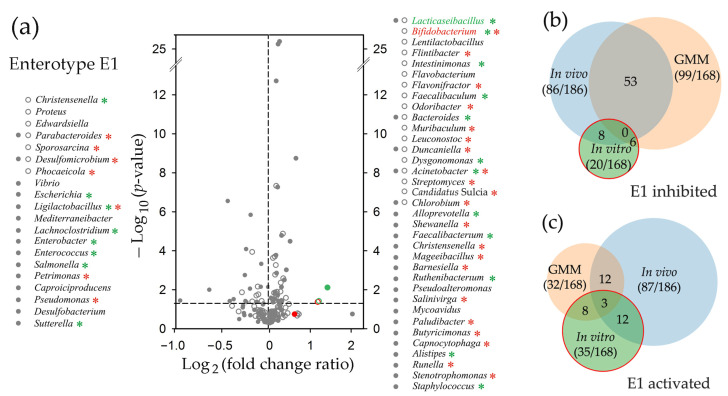
Model microbiomes of the E1 type cultivated ex vivo in GMM respond to probiotic intervention in vitro. (**a**) Probiotic-mediated changes were assessed following 24 h (open circles) and 48 h (filled circles) of cultivation. Volcano plot for 24 h samples was constructed using pairs: 1m_GM-1m_Pr, 5m_GM-5m_Pr, 6m_GM-6m_Pr, 6s_GM-6s_Pr, 7m_GM-7m_Pr and 12m_GM-12m_Pr. For 48 h samples, they were: 1m_GM*-1m_Pr*, 6m_GM*-6m_Pr*, and 6s_GM*-6s_Pr*. Red and green symbols correspond to bifidobacteria and lactobacilli, respectively. Asterisks indicate the genera that showed a similar (green) or an opposite (red) response in the E1 microbiomes in vivo (*p* < 0.05, dashed line). (**b**,**c**) Venn diagrams showing overlaps between the sets of inhibited (**b**) and activated (**c**) genera in response to probiotic administration in vivo (blue circles), addition of probiotic bacteria in vitro (green circles), and transition to GMM (peach circles). Numerals in parenthesis show the number of taxa with corresponding responses and the size of the set with pairs that passed the selective criteria. The other numbers indicate the size of the overlapping parts.

In the case of ex vivo cultured E1 biota (Figure 10), only seven genera were suppressed during the first 24 h of cultivation with probiotics (open circles), and two of them retained low abundance later (filled circles) when 13 additional taxa decreased their percentage. Most of the latter belong to the bacteria suppressed by probiotics in vivo (green asterisks in Figure 10a). However, this overlap is exactly what is expected by chance since 46.2% of the monitored genera were inhibited with *p* < 0.05 at different time points in the fecal biota in vivo (Figure 10b)**.** The set of bacteria that exhibited probiotic-mediated expansion included 12 taxa with similar responses in vivo (green asterisks in Figure 10a). However, this is even less than expected from the proportion (46.7%) of bacteria inhibited in vivo (Figure 10c), and the number of stimulated taxa with the opposite reaction in vivo was twice as large (red asterisks in Figure 10a). Thus, we did not find any evidence indicating similarity in the probiotic-mediated response of the E1 biota in vitro and in vivo.

**Figure 11:**
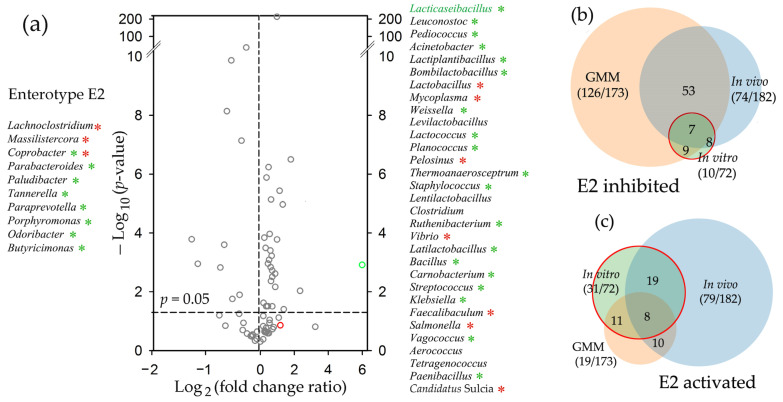
Probiotic-mediated rearrangements in the E2 microbiota in vitro showed similarities to the response of fecal E2 microbiomes to probiotic intervention in vivo. (**a**) Bacterial communities recovered from samples 3s, 7s, and 8s were cultured for 48 h in GMM with (samples 3s_Pr, 7s_Pr, and 8s_Pr) and without (samples 3s_GM, 7s_GM, and 8s_GM) the probiotic. The volcano plot shows probiotic-mediated changes in the E2 biota. Red and green symbols correspond to bifidobacteria and lactobacilli, respectively. Asterisks indicate the genera that showed a similar (green) or an opposite (red) response in the E2 microbiomes in vivo (*p* < 0.05, dashed line). (**b**,**c**) Venn diagrams show the overlap between the sets of inhibited (**b**) and activated (**c**) genera in response to probiotic administration in vivo (blue circles), the addition of probiotic bacteria in vitro (green circles), and the transition to GMM (peach circles). Numerals in parenthesis indicate the number of taxa with corresponding responses and the size of the set with pairs that met the selection criteria. The remaining numbers indicate the size of the overlapping parts.

In the revived microbiota of the E2 type, the outcome was different (Figure 11). Bacteria were cultured for 48 h with and without probiotics, and a statistically significant reaction was observed for 41 genera. Only ten taxa were suppressed (Figure 11a,b), which is likely a consequence of greater negative effects caused by environmental changes on the E2 microbiomes prior to probiotic application (Supplementary Figure S3). However, most of the inhibited bacteria belonged to genera that were also suppressed in vivo (green asterisks), which is twice more than expected by chance (Figure 11b). The number of taxa stimulated both in vitro and in vivo in the model E2 biota (Figure 11a,c) also turned out to be higher than expected (61.3% instead of 43.4%). Moreover, in contrast to the E1 revived biota, the proportion of genera exhibiting the opposite response to probiotic administration in vivo was much lower than that of similarly reacting taxa (0.29 and 0.37 for inhibited and stimulated bacteria, respectively). Thus, although the sets of probiotic-responsive taxa were small, their in vitro reactions partially reflected adaptive changes in the E2 microbiomes in vivo. Even the addition of probiotic bacteria to the E2 microbiomes, while increasing the percentage of lactobacilli, did not lead to the same increase in the abundance of bifidobacteria (Figure 11a), although the transfer to the laboratory medium was not suppressive for both probiotic genera (Supplementary Figure S3).

Therefore, we believe that it is possible to create media for bacterial communities derived from different natural microbiomes, which, while retaining the core of their bacterial consortia, will become relevant test systems specifically assessing the benefits of probiotics or the effectiveness of antibacterial drugs.

## 3. Discussion

The main goal of the study was to compare the response of the natural fecal biota to the introduction of two new isolates of *L. paracasei* and *B. longum* in vivo and in vitro, as well as to assess the feasibility of using simplified microbiomes extracted from the same feces as model communities for test systems. Rats were selected as a well-studied biological object prospective for assessment of gut microbiota shifts related to human pathological states [71]; however, in this study, we chose to use only male animals in hopes of obtaining microbiomes that are less dependent on individual variability caused by estrous cycles. GMM medium suggested by Goodman et al. [72] was implemented because it provides a possibility to capture a remarkable proportion of natural fecal microbiota using anaerobic culturing conditions and easily available reagents [72]. Since different representatives of lactobacilli and bifidobacteria are often used together in probiotic compositions, in this study, we also combined them in equal proportions (about 10^8^ CFU) and did not use these species separately. This permitted us to compare relative changes in the abundance of bacteria belonging to corresponding genera. As a result, we found that the intervention of *B. longum* as part of probiotic consortia reproducibly reduced the percentage of bifidobacteria in E2 biota with a parallel increase in the abundance of lactobacilli (Figure 5b). Being partly consistent with the observation made by Haddad et al. [55], who did not observe an increase in the presence of bifidobacteria and lactobacilli after their combined administration in humans, our data are more informative because only adaptive aggressive reaction of resident microbiota can explain decrease in the number of alive bacteria (Figure 6b). To our knowledge, this is the first observation of a negative reaction from resident microflora on probiotic bacteria.

Consistent with the results obtained in [60], we identified two enterotypes in the gut biota of male rats. However, both differed from previously published phyla in the relative abundance of *Bacteroidetes* (now *Bacteroidota*) and *Firmicutes* (now *Bacillota*), which is one of the discriminatory criteria used in taxonomic analysis. Since Kalinski-Harabasz indices computed to determine the optimal number of clusters for some smaller sets of marker taxa indicated the advisability of clustering into three and even five groups, and Choi et al. [60] predicted three enterotypes for female rats, it is possible that rat microbiomes are more diverse. If this is the case, then the microbiomes characterized in these two studies may represent four different types, as suggested for the gut microbiomes of 2-year-old rats by Lee et al. [73]. Unfortunately, datasets generated from different sequencing platforms and targeting different 16S rRNA variable regions cannot be used for co-clustering. In any case, enterotypes observed in rats were highly similar to those typical for the human gut [44]. Although there are some non-conformities that may be attributed to a variety of differences in the human and rat ecology, diet, and metabolism, *Firmicutes* and *Bacteroidetes* dominated in both cases in different mutual ratios [44,60,73,74]. As an example, of the 30 most abundant genera identified in different types of human microbiomes [44], 18 were selected for the set of 208 traceable taxa based on their high presence in rat samples, and 16 (88.9%) of them were activated or inhibited in response to probiotic administration in vivo (Figure 4a and Figure 5b) in an enterotype-dependent manner. *Blautia*, for instance, was inhibited only in the E2 biota; *Akkermansia* (*Verrucomicrobia*, now *Verrucomicrobiota*) and *Acidaminococcus* were activated only in the E1 microbiomes, while *Eubacterium* showed an opposite reaction in two enterotypes. Thus, it is likely that the successful “humanization” of the sterile rat intestine by the human microbiome [71] is due to the inherent similarity of at least the dominant genera in the guts of rats and humans.

In this study, we traced three types of taxonomic shifts in microbiomes. These include the response of the bacterial community to probiotic administration in vitro and in vivo, as well as its transfer to the laboratory medium. In all cases, we observed a reaction with a clear dependence on the enterotype. It was manifested not only in different compositions of probiotic-responsive genera but also in the presence of taxa that showed opposed responses to the probiotic introduction in the E1 and E2 microbiomes (red asterisks in Figure 4a and Figure 5b). This means that the homeostasis of each of the two enterotypes responded to the intervention as an integral system, and a representative set of oppositely responding genera (red asterisks in Figure 4a and Figure 5b) could potentially be used as enterotype markers instead of traditionally used specification by one dominant genus/phylum [44].

Complete enterotyping is a non-trivial task since it requires a profound in silico clustering with a large number of tracked taxa and may be changed by diet [75,76], antimicrobial therapy [77], or even probiotic intervention (Figure 8a). Thus, for example, using a silhouette validation approach to assess the robustness of clustering, we observed a clear shift of the E1 microbiomes towards the E2 type after the third dose of probiotic intake (Figure 8a), which was almost restored eleven days later (Sample 59 in Figure 8a), but this is not obvious from the pie charts in Figure 4b. It is not yet clear to what extent the response of microbiomes to external factors is determined by the enterotype, i.e., the integral state of the entire network of interspecies interactions in the microbiota or specific stimulation/suppression of symbiotic/antagonistic relationships between individual members of the bacterial community plays the major role in triggering this process. The answer to this question may be the subject of future research, and our discovery of enterotype switching by simple addition of probiotic bacteria can be used for planning such studies.

There are several other approaches for developing model systems to assess the influence of external factors, including bacterial species, on the taxonomy of target microbiomes, and artificial microbiomes are considered a promising way to create standard test systems [65,66,67] that can be specifically designed for different enterotypes. An alternative way, which in some respects may be more relevant for practical use, is to develop strategies for monitoring the response of natural microbiomes in reduced communities individually extracted from biological samples. Our data indicate that the homeostasis of fecal biota transferred to GMM was stable enough to support non-overlapping taxonomic clustering (Figure 9), and samples with the E2 enterotype (Figure 11) responded similarly to probiotic intervention in vitro and in vivo, substantiating the hope for the practical usefulness of further efforts in this direction. In addition to the advantage due to the individuality of the tested samples, this also makes it possible to retain at least some of the uncultured species in the bacterial communities. In the human intestine, the number of such microbes totals up to 70% [78], which indicates their high significance in the metabolic balance of colonizing bacteria. Since many uncultured species can grow in mixed communities [79,80,81], culturing natural microbiomes ex vivo can preserve their contribution to the overall homeostasis, i.e., to provide more apt models for screening.

Taken together, we believe that the following observations made in this study significantly contributed to the field. (1) The enterotype of the gut microbiota may transiently change in response to repeated probiotic administration. (2) Probiotic bacteria do not necessarily exhibit expansion in the resident biota. (3) Natural microbiomes transferred to a laboratory medium can be used to assess individual responses to probiotics or antimicrobials.

## 4. Materials and Methods

### 4.1. Bacterial Strains, Growth Conditions and Lyophilization

New isolates of *Bifidobacterium longum* subsp. *longum* 202_21 (*B. longum*) and *Lacticaseibacillus paracasei* subsp. *paracasei* 29n_21 (*L. paracasei*) were specified using 16S rRNA phylotyping with primers 5′-AGAGTTTGATCCTGGCTCAG-3′ (27f) and 5′-TACCTTGTTACGACTT-3′ (1492r) (Evrogen, Moscow, Russia). For in vitro experiments, isolates were individually cultivated for 48 h at 37 °C in Hungate tubes containing 12 mL of MRS medium deoxygenated by purging for 15 min with a gas mixture of 80% N_2_ and 10% CO_2_. MRS medium contained (per liter): peptone, 10.0 g; meat extract, 10.0 g; glucose, 20.0 g; yeast extract, 5.0 g; Tween-80, 1.0 g; ammonium citrate, 2.0 g; sodium acetate, 1.0 g; potassium hydrogen phosphate, 2.0 g; MgSO_4_ × 7H_2_O, 12.0 mg; MnSO_4_ × 7H_2_O, 0.05 g; L-cysteine HCL, 0.5 g; resazurin, 0.1 mg.

For the feeding experiment in vivo, bacteria were cultivated under anaerobic conditions in an atmosphere of a three-component gas mixture of 80% N_2_, 10% CO_2_, and 10% H_2_ on solid media (1.5% agar) at a temperature of 37 °C for 48 h. *L. paracasei* was cultivated on an MRS medium, while for *B. longum,* a TOS propionate milieu specifically optimized for bifidobacteria was used. It included (per liter) sodium propionate, 15.0 g; ammonium sulfate, 3.0 g; casein peptone, 10.0 g; yeast extract, 10.0 g; galactooligosaccharide TOS, 1.0 g; L-Cysteine hydrochloride, 10.0 g; disubstituted potassium phosphate, 0.5 g; MgSO_4_ × 7H_2_O, 4.8 g; monosubstituted potassium phosphate, 0.2 g. Colonies of both strains were collected from the agar by cotton swabs in a cell count of 2.1–2.4·10^8^ CFU (3 therapeutic doses) and placed in the vials with 20% sterile deoxygenized milk with 0.025 μg/mL of lacto-N-biose (C_14_H_25_NO_11_) or N-acetylglucosamine. The vials were sealed with a sterile, non-absorbent cotton stopper and placed in a freezer (−70 °C) for 16 h. They were then transferred to a chilled freeze dryer and dried. The samples were homogenized in a laminar flow hood, and the cotton stoppers were replaced with airtight lids.

To determine the titer of live bacteria, 10 mL of Luria-Bertani medium (tryptone 1.0 g/L, yeast extract 0.5 g/L, and NaCl 1.0 g/L) were added with a syringe to a vial containing lyophilized bacteria. After 30 min of suspension at 37 °C, serial dilutions (10^−^^2^, 10^−^^3^, 10^−^^4,^ and 10^−^^5^) were prepared in Hungate tubes with either MRS media (preferential cultivation of lactobacilli) or TOS propionate milieu with the addition of muporacine (0.025 g/L) to inhibit the growth of lactobacilli. Bacteria taken in 1 mL were grown in an anaerobic jar (72 h at 37 °C) in triplicate after their surface plating (1 mL) on solid MRS media in Petri dishes (only *L. paracasei* grew) and after their “deep seeding” in 15 mL of TOS-MUP medium (Figure 6b,c).

### 4.2. Animal Handling and Administration of Probiotics In Vivo

Experiments were conducted with healthy SPF-grade male Wistar rats weighing 160–200 g. Rats were bred in animal facility “Pushchino” in accordance with the Directive 2010/63/EU of the European Parliament and of the Council of 22 September 2010 on the protection of animals used for scientific purposes (Protocol 21-08PZ#V3 of 29 June 2021 “Conducting preclinical studies of toxicity from single injection and efficiency of finished dosage form of next-generation probiotics”). Following adaptation with improved conventional status, the animals were occasionally split into two groups, and four animals were kept per cage with a 12-h day and night cycle. Animals were fed ad libitum with permanent access to fresh water. Rats received 1 mL suspension of lyophilized probiotic bacteria in phosphate buffer with magnesium chloride as a single dose (group “single”) or three doses (group “multiple”) at daily intervals (Figure 1). Suspensions were prepared in sterile conditions immediately prior to application. Fecal samples were collected individually from 9 animals of the first group and 12 rats of the group with multiple feeding on days 1 (control samples), 2, 3, 4, and 14 relative to the treatment, placed in individual sterile plastic bags and frozen at −40 °C until usage.

### 4.3. Revival of Fecal Bacteria

Rat fecal samples (0.1–0.2 g) were homogenized in 1 mL of gut microbiota medium (GMM), as suggested in [72]. The medium containing: tryptone peptone, 0.2%; yeast extract, 0.1%; D-glucose, 2.2 mM; L-cysteine, 3.2 mM; cellobiose, 2.9 mM; maltose, 2.8 mM; fructose, 2.2 mM; meat extract, 0.5%; KH_2_PO_4_, 10 mM; MgSO_4_ × 7H_2_O, 0.008 mM; NaHCO_3_, 4.8 mM; NaCl, 1.37 mM; Tween-80, 0.05%; CaCl_2_, 0.072 mM; resazurin, 4 µM; vitamin K, 5.8 µM; FeSO_4_ × 7H_2_O, 1.44 µM; histidine-hematin stock solution, 0.1% (1.2 mg/mL hematin in 0.2 M histidine), vitamin mix, 0.1%; trace mineral mix, 0.1%, pH 7.0) was mixed by vortexing with silica beads. GMM preparation was as follows: all the components (except vitamin K, FeSO_4_ × 7H_2_O, histidine-hematin, vitamin mix, and trace mineral mix, which were filter-sterilized) were dissolved, carefully heated, cooled under CO_2_ flow, transferred to N_2_-purged Hungate tubes, tightly closed, and sterilized 20 min at 121 °C in an autoclave. Insoluble particles were carefully pelleted using a spinner. The supernatant was collected using a sterile syringe and transferred to Hungate tubes with 10 mL GMM. The inoculated medium was cultured for 48 h at 37 °C with slight agitation. Then the entire volume of the obtained culture was divided in halves and transferred to Hungate tubes containing an equal volume of GMM. One of the two samples of the pair was then supplemented with a mixture of *B. longum* and *L. paracasei* taken as 1/20 of the total culture volume.

### 4.4. Probiotic Administration In Vitro

Freshly prepared probiotic bacteria grown in MRS at 37 °C in anaerobic conditions were pelleted by centrifugation (20 min, 3000 rpm), dissolved in the required volume of GMM, and mixed in equal proportions. They were added to ex vivo-cultured microbiomes at a final titer of 0.65–1.1 × 10^8^ CFU. The growth of artificial microbiomes was carried out in Hungate tubes under anaerobic conditions at 37 °C, and cells were collected at two time points: 24 and 48 h.

### 4.5. DNA Extraction

QIAamp^®^ Fast DNA Stool Mini Kit (Qiagen, Hilden, Germany) was used for DNA extraction from fecal samples according to the manufacturer’s instructions for the isolation of DNA from pathogenic bacteria. Elution of DNA solutions from filters was performed by sequential application of two 30 µL portions of nuclease-free water. The DNA samples obtained were cleaned additionally with Monarch^®^ PCR & DNA Cleanup Kit (New England BioLabs, Ipswich, MA, USA). Nuclease-free water was used for elution. Ex vivo-cultured bacteria were harvested by centrifugation (20 min, 3000 rpm), washed with sterile PBS, and processed for DNA isolation using QIAamp Fast DNA Stool Mini Kit (Qiagen, Germany). The concentration of DNA in both cases was measured on a Qubit 3.0 fluorometer (Life Technologies, Thermo Fisher Scientific, Waltham, MA, USA). The content of impurities was checked through 260/280 nm and 260/230 nm ratios using a Nanodrop ND-1000 spectrophotometer (Thermo Scientific, Wilmington, DE, USA) to ensure that these parameters are not below 2.0 and 1.8 values, as considered critical for nanopore sequencing library preparation by Oxford Nanopore Technologies (Oxford, UK).

### 4.6. 16S Ribosomal RNA Gene Amplification

PCR amplification was performed in the volume of 50 µL on a Veriti 96-well Thermal Cycler (Applied Biosystems, Thermo Fisher Scientific, Foster City, CA, USA) with standard primers 27f and 1492r (Evrogen, Russia) and Q5 High-Fidelity DNA Polymerase (New England BioLabs, Ipswich, MA, USA). Amplicons were purified from the reaction components with AMPure XP magnetic beads (Beckman Coulter, Brea, CA, USA). DNA samples were diluted to 20 ng/µL before amplification, and 1 µL per reaction was used as a template. Amplification program: 95 °C, 5 min; 30 cycles—(95 °C, 1 min, 59 °C, 1 min, 72 °C, 1 min 30 s); 5 min 72 °C. Two microliters of amplicons were sampled to run in 1% agarose gel to test the efficiency of amplification. Then, amplicons were cleaned with AMPure XP Magnetic Beads (GE Healthcare, Chicago, IL, USA) as described in Oxford Nanopore Technologies SQK-LSK109 protocol.

### 4.7. DNA Library Preparation and Sequencing

The DNA libraries was prepared according to SQK-LSK109 Ligation sequencing gDNA kit (Oxford Nanopore Technologies, Oxford, UK) with EXP-NBD104 and EXP-NBD114 barcode expansions (Oxford Nanopore Technologies, Oxford, UK) according to the protocol of the manufacturer with minor changes. The stage of DNA repair with NEBNext^®^ FFPE DNA Repair Mix (New England Biolabs, Ipswich, MA, USA) and NEBNext^®^ Ultra™ II End Repair/dA-Tailing Module (New England Biolabs, Ipswich, MA, USA) was extended to 25 min, and ligation—to 60 min. Other stages remained unchanged. Sequencing was performed on the MinION Mk1B platform (Oxford Nanopore Technologies, Oxford, UK) with R9.4.1 flow cells. Sequencing data statistics for all samples are available in Supplementary Table S3. Basecalling was carried out in High-Accuracy mode with a threshold of 7 (MinKNOW GPU 23.04.3). 

### 4.8. Identification of Taxa for Clustering

All datasets with reads were classified using EPI2ME v. 6.3.2 software and the Fastq WIMP v2023.06.13-1865548 algorithm. Bacterial genera with an abundance of 0.01% and higher were selected in each dataset. The genera lists were then combined, and 208 taxa with indicated abundance in at least one data set were collected. The number of reads and their percentages in each set were calculated for all 208 marker taxa. Their profiles appeared to be highly consistent within experimental groups (available in the NCBI SRA Database, accession number PRJNA1039726). Outliers were detected only in 3 out of 94 sequencing libraries (*Mycobacterium*, *Escherichia,* and *Nostoc* in datasets 3s, 37m, and 57s, respectively). Their contributions were ignored, and the percentage of the other 207 taxa was calculated based on the total number of the remaining reads in the set.

### 4.9. Enterotype Assessment and Visualization

Major parts of analysis and visualization were carried out with software package R v. 4.3.0 using the following libraries: cluster, clusterSim, ade4, ggforce, ggplot2, patchwork, scales, tidyverse, phyloseq, microbiomeMarker. First, the CSV datasets with comma-separated values were processed with R script to convert them into row-column format, and the percentage of all tracked taxa was calculated. Clustering of the datasets was performed as in [44] with a slightly modified R script provided at https://enterotype.embl.de/enterotypes.html (assessed 17 April 2024). In brief: Jensen-Shannon distances between datasets were calculated based on different in size and composition sets with marker taxa (from 46 genera collected as top 20 dominant taxa in control samples to 208 genera with at least a single presence in percentage > 0.01 in any set). The results of the computations were used in partition around medoids clustering (PAM). The Calinski–Harabasz indices computed at this stage revealed variability in the optimal number of potential clusters. Since the 208 genera set suggested partitioning into two groups, as it was reported for microbiomes of male rats’ intestines [60], final clustering in all series was made into two groups (Figure 2, Figure 6 and Figure 7). To evaluate the accuracy and reliability of clustering, silhouette scores of all fecal samples were estimated for all data sets obtained at different time points, which were combined in 10 possible pairs, ten triplets, five quartets, and one quintet (Figure 7b and Figure 8a). For silhouette scores above 0.1, the clustering result for the sample was considered true. The number of true assignments for each sample in both clusters was then calculated, and the final decision was made based on their number. This confirmed the automatically predicted clustering and enterotype shift at the 72-h time point.

### 4.10. Volcano Plots Visualization

Volcano plots were prepared based on Z-scores calculated for fold change ratios (FCR) in the abundance of tracked genera. Samples of two enterotypes were assessed independently. First, the percentage values of all 208 tracked genera were estimated in all 94 data sets. To avoid dividing by zero, one read was added to genera without robotically assigned reads. In the feeding experiment (Figure 4a and Figure 5b), FCRs were calculated for experimental samples obtained at different time points and control metagenomes obtained from animals before probiotic administration. The same control samples were used to assess rearrangements caused by the transition of fecal microbiota to GMM (Supplementary Figures S2 and S3). In the case of in vitro probiotic administration, samples obtained from ex vivo-cultured bacterial consortia grown in parallel with the same cultures supplemented with a probiotic were compared (Figure 10a and Figure 11a). Since the percentages of bifidobacteria and lacticaseibacilli in sample 5m_GM* were significantly higher than in the other samples of this group (1m_GM*, 6m_GM*, and 6s_GM*), it is possible that samples 5m_GM* and 5m_Pr* were confused. Therefore, to avoid any bias, we excluded the 5m–5m_GM* and 5m_GM*–5m_Pr* pairs from the samples used for the volcano plots in Supplementary Figure S2 and Figure 10a, respectively. To avoid inconsistency due to the small number of reads assigned to some genera, only those paired samples for which the percentage in the experimental sample with increased abundance was ≥0.01 and those suppressed taxa for which the percentage in the control sample was ≥0.01 were used. If there were at least three paired specimens meeting these criteria in the available data sets, the genus was included in the list of taxa assessed. Their numbers for different groups are indicated in Venn diagrams (Figure 10b and Figure 11b). FCRs were then averaged within groups, StD values were estimated, and Z-scores were calculated in Excel using FCR = 1 as the null hypothesis parameter. For Z-scores less than 8, *p* values were automatically calculated in Excel using the 1-NORM.S.DIST(Z; TRUE) option. For larger Z-scores, the *p*-values were estimated individually in the R software package.

### 4.11. Pie Charts Visualization

Candidates for dynamic change visualization were selected from 108 genera that demonstrated at least a 2-fold statistically significant alteration in at least one of seven time points and met the criteria of Section 4.11 in all compared data sets. To reduce the proportion of normalization insertions (gray sectors in Figure 4a and Figure 5b) required to balance the overall percentage of chosen taxa at different time points, we selected genera based on their abundance in samples rather than based on FCR. To balance the contribution of inhibited and stimulated taxa in the E1 and E2 microbiomes, genera were collected in a stepwise manner. Thus, to the dominant taxon in the sample with the control E1 biota, from the remaining 107 genera, the genus with the maximum presence in the control sample with biota of type E2 was first added. The most abundant taxa from the remaining 106 and 105 genera were then selected from the microbiomes of rats (E1 and E2, respectively) given one dose of probiotics, and so on for other time points (48 h, 72 h, and 14 days). Since the dynamics of changes in the E1 microbiome throughout the entire feeding experiment were traced only for one rat (sample 11m), the selection procedure was carried out using a combination of its microbiome with microbiomes obtained from control samples E2 1s, 2s, and 9s. Representative genera collected in combination 11m-1s showed the lowest dependence in the total percentage from the time points and were used for pie chart visualization. Fifty representative taxa were collected via five consecutive rounds of selection in the order: 11m-1s-23m-13s-35m-25s-47m-37s-59m-49s. Finally, the dominant *Muribaculum* and *Prevotella* in the 11m and 1s control sets, with total contributions of 15.4% and 12.6%, respectively, were removed from the set to increase the resolution for other taxa in the pie chart.

### 4.12. Biodiversity Assessment

Taxonomic analysis for alpha diversity estimation was performed with wf-metagenomics Nextflow workflow (https://github.com/epi2me-labs/wf-16s, accessed on 17 April 2024) using minimap2 [82] and the NCBI 16S/18S rRNA database. Shannon indices were calculated with the sk.diversity.alpha.shannon function from skbio python library.

### 4.13. Venn Diagrams

To estimate an overlap between different sets of taxa reacting to the probiotic administration in vitro and in vivo, as well as with sets responding to stress caused by environmental change, all genera with statistically significant changes (*p* < 0.05) were used. Genera responding in a time- or dose-dependent manner were combined, and the intersection between groups was estimated using the sorting options of Excel v. 15.0.5603.1000. Venn diagrams in Figure 8 and Figure 9 were created using an online Venn diagram maker at https://www.meta-chart.com/venn#/display accessed on 17 April 2024.

### 4.14. Statistics

For bar plots in Figure 6 and Supplementary Figure S1, *p*-values for compared sets were estimated based on Z-scores using FCR of paired samples as input sets and R = 1 as the null hypothesis. The One-Sample *t*-test in SigmaPlot v. 11 (https://grafiti.com, accessed on 17 April 2024) was implemented to check normality in the distribution of numerical values. The “Compare Two Groups” option in the same package was implemented to estimate the *p*-value of differences between two groups of numerical values (number of identified taxa and Shannon index of biodiversity) belonging to the E1 and E2 enterotypes.

## Data Availability

The datasets presented in this study are available at https://dataview.ncbi.nlm.nih.gov/object/PRJNA1039726.

## Supplementary Materials

Supporting information can be downloaded at https://www.mdpi.com/article/10.3390/ijms25084558/s1.
