# Supplementary material for: Enterotype-Dependent Probiotic-Mediated Changes in the Male Rat Intestinal Microbiome In Vivo and In Vitro
Source: Int J Mol Sci. 2024 Apr 22;25(8):4558. doi: 10.3390/ijms25084558 (PMC11049970; doi:10.3390/ijms25084558)
Supplement: Supplementary file 1 [file ijms-25-04558-s001.zip › Supplementary Figure S1.pdf]

Supplementary Figure S1

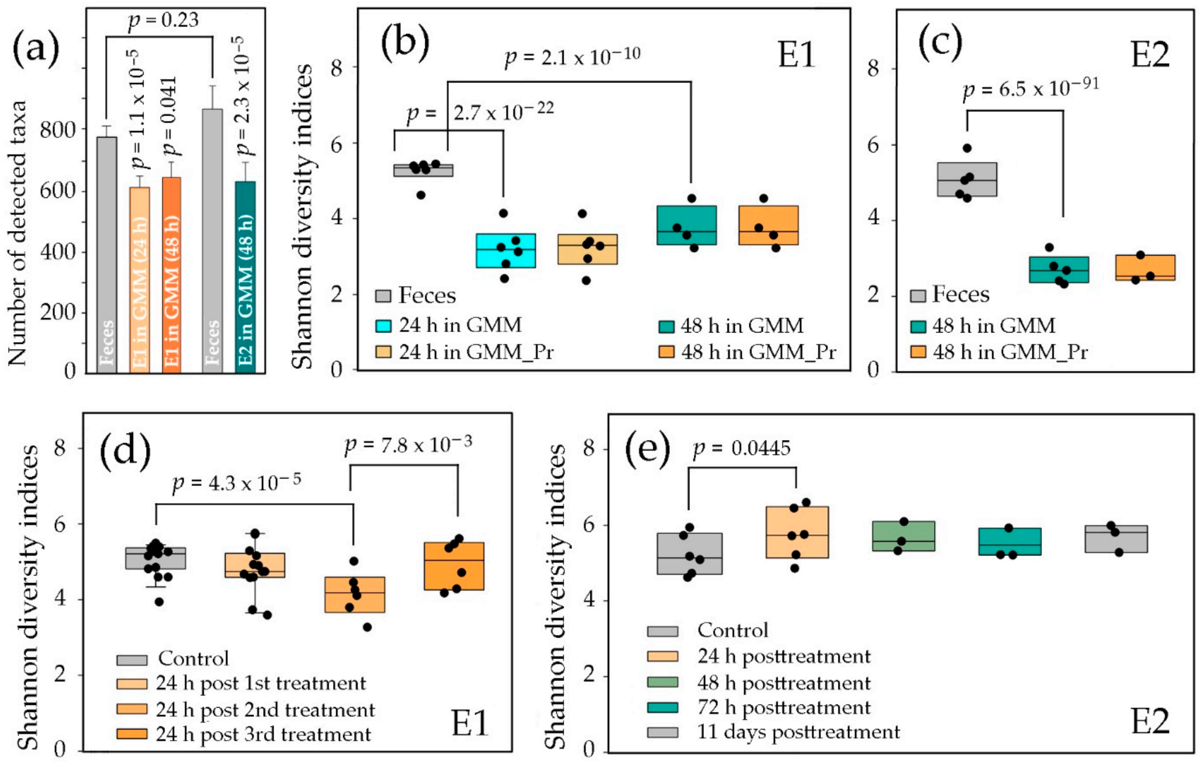

Figure S1. Variation in the number of detected taxa and biodiversity of microbiomes obtained from feces and *ex vivo* cultured bacterial communities. (**a**) The number of identified taxa in fecal samples 1m, 5m – 7, 6s, 12m (E1) and 1s, 3s, 7s – 9s (E2) was not statistically different and decreased to approximately the same values in samples cultured *ex vivo* for 24 or 48 hours. (**b,c**) Transferring the aforementioned fecal microbiomes to GMM significantly reduced Shannon diversity indices (H'). In the E1 samples 1m_GM, 5m_GM – 7m_GM, 6s_GM and 12m_GM cultured 24 hours, H' decreased from 5.23 ± 0.17 to 3.20 ± 0.24 and increased to 3.78 ± 0.28 (sample pairs 1m-1m_GM*, 5m-5m_GM*, 6m-6m_GM* and 6s-6s_GM*) after 48 h cultivation. In the E2 samples 1s_GM*, 3s_GM*, 7s_GM* – 9s_GM*, cultured for 48 hours, H' decreased from 5.08 ± 0.23 to 2.70 ± 0.17, providing a statistically significant difference (p = 0.01) between the reactions of two enterotypes. The addition of the probiotic did not affect the biodiversity of *ex vivo* cultured bacteria (**b,c**). However, after the 2-nd dose of the probiotic (samples 25m – 28m, 33m, 35m, 28s), a temporary decrease in the biodiversity of the E1 microbiomes was observed compared to samples 1m – 4m, 4s, 9m, 11m (**d**), which was also accompanied by a statistically significant increase from H' = 4.28 ± 0.24 to 4.95 ± 0.26 in samples 37m – 40m, 45m, 47m (p = 0.05) (**d**). The single administration of the probiotic in rats with the E2 biota (samples 1s – 3s, 7s – 9s), caused an average increase for 11.5 ± 6.7% (samples 13s – 15s, 19s – 21s), rather than a decrease in H' typical for the E1 microbiomes. This was not statistically significant (**e**), but provided further evidence of the differential response of the two enterotypes to probiotic exposure.
