# Supplementary material for: Enterotype-Dependent Probiotic-Mediated Changes in the Male Rat Intestinal Microbiome In Vivo and In Vitro
Source: Int J Mol Sci. 2024 Apr 22;25(8):4558. doi: 10.3390/ijms25084558 (PMC11049970; doi:10.3390/ijms25084558)
Supplement: Supplementary file 1 [file ijms-25-04558-s001.zip › Supplementary Figure S2.pdf]

Supplementary Figure S2

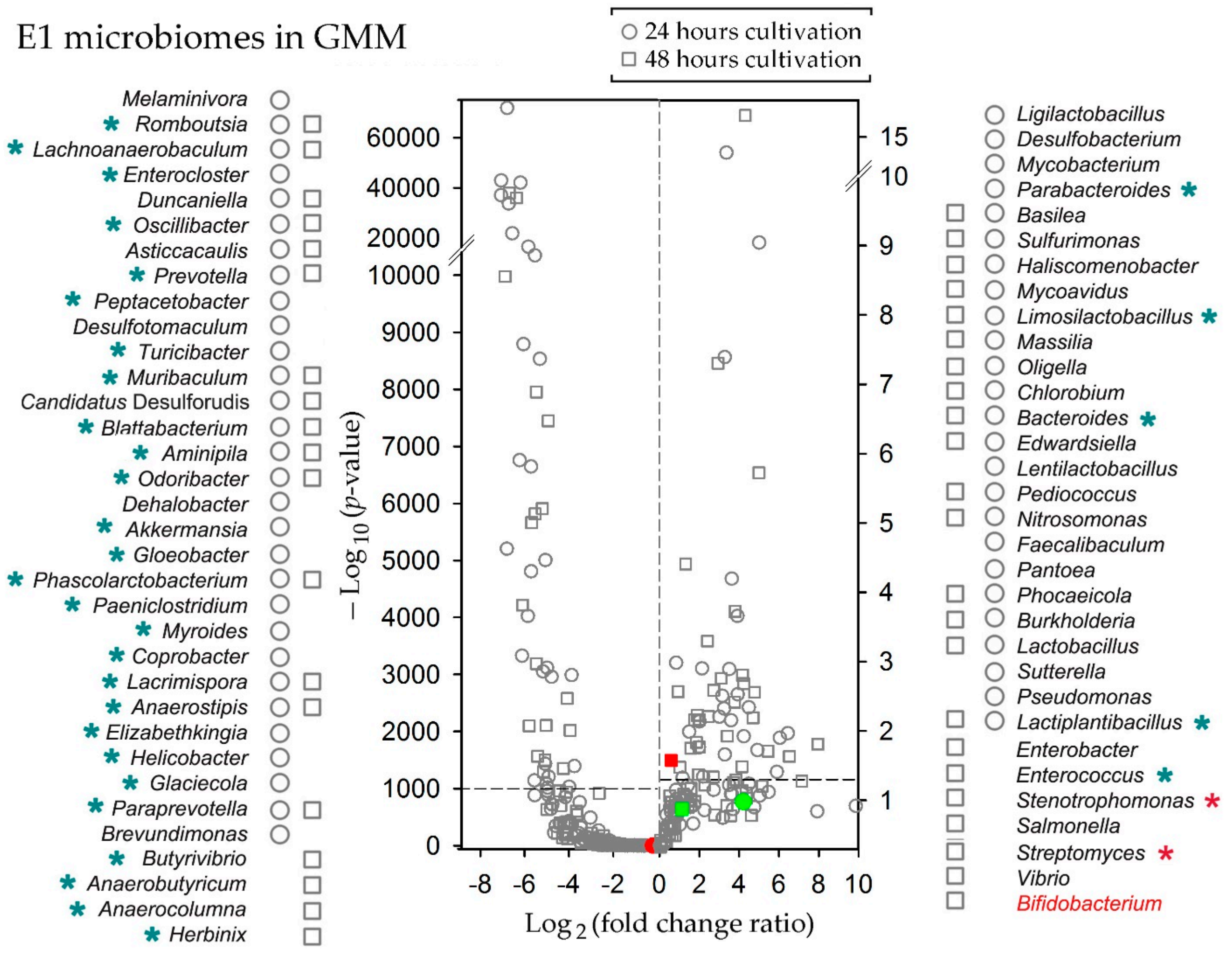

Figure S2. Transfer of the E1 fecal biota to GMM significantly ($p < 0.05$) suppressed 99 tracked genera and activated the growth of 32 taxa. A total of 168 genera met the selection criteria for comparative evaluation. The volcano plot was constructed using paired samples 1m–1m_GM, 5m–5m_GM, 6m–6m_GM, 7m–7m_GM, 6s–6s_GM and 12m–12m_GM (open circles), as well as pairs 1m–1m_GM*, 6m–6m_GM*, 6s-6s_GM* (rectangles) (**Figure 1**). Red and green symbols correspond to bifidobacteria and lacticaseibacilli, respectively. Genera with negative (left) and positive (right) reactions are listed in a descending order of effect caused by the changed environment. Only those taxa whose negative decimal logarithms of p-values exceeded the thresholds indicated by the dashed lines were included in the lists. The symbols near the taxa names correspond to the time of bacteria cultivation in the GMM. Asterisks mark genera with similar (cyan) or opposite (red) response in the E2 microbiomes ($p<0.05$).
