# Supplementary material for: Enterotype-Dependent Probiotic-Mediated Changes in the Male Rat Intestinal Microbiome In Vivo and In Vitro
Source: Int J Mol Sci. 2024 Apr 22;25(8):4558. doi: 10.3390/ijms25084558 (PMC11049970; doi:10.3390/ijms25084558)
Supplement: Supplementary file 1 [file ijms-25-04558-s001.zip › Supplementary Figure_S3.pdf]

Supplementary Figure S3

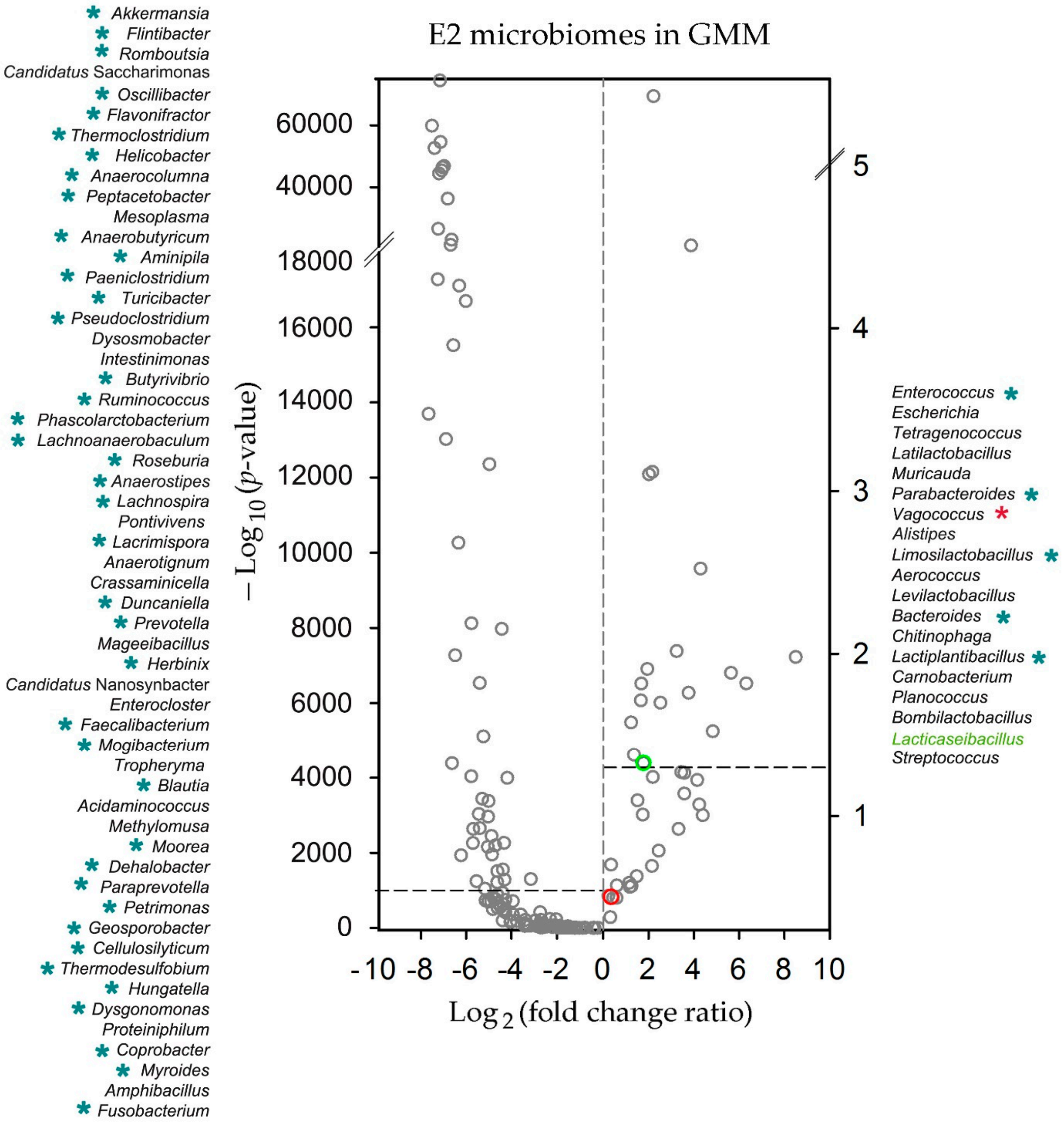

Figure S3. Transfer of the E2 fecal biota to MM and culturing during 48 hours significantly ($p < 0.05$) suppressed 126 tracked genera and activated the growth of 19 taxa. A total of 173 genera met the selection criteria for comparative evaluation. The volcano plot was constructed using paired samples 1s–1s_GM*, 3s–5s_GM*, 7s–7s_GM*, 8s–8s_GM* and 9s–9s_GM* (**Figure 1**). Red and green symbols correspond to bifidobacteria and lactobacilli, respectively. Genera with negative (left) and positive (right) reactions are listed in a descending order of effect caused by the changed environment. Only those taxa whose negative decimal logarithms of p-values exceeded the thresholds indicated by the dashed lines were included in the lists. Asterisks mark genera that showed a similar (cyan) or opposite (red) response in the model E1 microbiome ($p<0.05$).
