# Supplementary material for: Enterotype-Dependent Probiotic-Mediated Changes in the Male Rat Intestinal Microbiome In Vivo and In Vitro
Source: Int J Mol Sci. 2024 Apr 22;25(8):4558. doi: 10.3390/ijms25084558 (PMC11049970; doi:10.3390/ijms25084558)
Supplement: Supplementary file 1 [file ijms-25-04558-s001.zip › Supplementary Table S3.pdf]

Table S3. Sequencing data statistics for 16S rRNA amplicons of rat fecal microbiomes

Sample name	Number of reads classified by WIMP	Number of taxa identified	Notes
Single probiotic administration			
1s	412,314	1,044	Control
2s	387,546	1,037	Control
3s	348,957	726	Control
4s	357,285	591	Control
5s	401,905	663	Control
6s	162,361	626	Control
7s	79,039	768	Control
8s	209,944	916	Control
9s	156,105	833	Control
13s	715,583	1,163	Following 24 h after feeding
14s	272,461	978	Following 24 h after feeding
15s	105,860	855	Following 24 h after feeding
16s	369,737	624	Following 24 h after feeding
17s	475,914	658	Following 24 h after feeding
18s	738,178	882	Following 24 h after feeding
19s	80,383	792	Following 24 h after feeding
20s	89,838	760	Following 24 h after feeding
21s	179,526	832	Following 24 h after feeding
25s	851,062	1,177	Following 48 h after feeding
26s	439,167	1,070	Following 48 h after feeding
28s	483,084	679	Following 48 h after feeding
33s	489,578	1,048	Following 48 h after feeding
37s	457,789	1,064	Following 72 h after feeding
38s	132,651	777	Following 72 h after feeding
41s	459,653	691	Following 72 h after feeding
45s	463,060	1,013	Following 72 h after feeding
49s	365,947	1,088	Following 14 days after feeding
50s	59,116	663	Following 14 days after feeding
52s	537,726	656	Following 14 days after feeding
53s	454,820	728	Following 14 days after feeding
57s	505,977	995	Following 14 days after feeding
1s_48 h_GM	371,712	770	Control after 48 h outgrowth in GMM
3s_48 h_GM	301,319	603	Control after 48 h outgrowth in GMM
3s_48 h_Pr	192,838	542	Following 48 h outgrowth with probiotic
6s_24 h_GM	424,186	562	Control after 24 h outgrowth in GMM

6s_24 h_Pr	221,242	460	Following 24 h outgrowth with probiotic
6s_48 h_GM	232,389	565	Control after 48 h outgrowth in GMM
6s_48 h_Pr	267,744	583	Following 48 h outgrowth with probiotic
7s_48 h_GM	149,138	424	Control after 48 h outgrowth in GMM
7s_48 h_Pr	163,048	523	Following 48 h outgrowth with probiotic
8s_48 h_GM	206,810	569	Control after 48 h outgrowth in GMM
8s_48 h_Pr	150,803	601	Following 48 h outgrowth with probiotic
9s_48 h_GM	321,719	761	Control after 48 h outgrowth in GMM
**Multiple probiotic administration**			
1m	658,171	721	Control
2m	603,233	812	Control
3m	849,834	732	Control
4m	1,410,558	831	Control
5m	547,532	787	Control
6m	769,544	870	Control
7m	449,313	835	Control
8m	1,251,954	773	Control
9m	412,215	749	Control
10m	803,606	741	Control
11m	379,094	737	Control
12m	424,142	802	Control
13m	577,356	652	Following 24 h after feeding
14m	206,977	591	Following 24 h after feeding
15m	264,550	747	Following 24 h after feeding
16m	87,948	559	Following 24 h after feeding
17m	1,155,941	892	Following 24 h after feeding
18m	1,376,484	1,115	Following 24 h after feeding
19m	940,402	831	Following 24 h after feeding
21m	218,470	621	Following 24 h after feeding
23m	161,349	619	Following 24 h after feeding
24m	698,270	854	Following 24 h after feeding
25m	444,933	593	Following 48 h after feeding
26m	202,506	648	Following 48 h after feeding
27m	101,139	589	Following 48 h after feeding
28m	222,208	729	Following 48 h after feeding
33m	247,657	656	Following 48 h after feeding
35m	377,733	569	Following 48 h after feeding
37m	668,193	984	Following 72 h after feeding
38m	326,271	971	Following 72 h after feeding
39m	172,977	791	Following 72 h after feeding

40m	134,643	733	Following 72 h after feeding
45m	437,903	1,017	Following 72 h after feeding
47m	380,901	929	Following 72 h after feeding
59m	520,027	644	Following 14 days after feeding
1m_24 h_GM	397,031	648	Control after 24 h outgrowth in GMM
1m_24 h_Pr	422,797	649	Following 24 h outgrowth with probiotic
1m_48 h_GM	257,683	635	Control after 48 h outgrowth in GMM
1m_48 h_Pr	293,448	658	Following 48 h outgrowth with probiotic
5m_24 h_GM	470,880	620	Control after 24 h outgrowth in GMM
5m_24 h_Pr	1,138,929	676	Following 24 h outgrowth with probiotic
5m_48 h_GM	484,348	566	Control after 48 h outgrowth in GMM
5m_48 h_Pr	600,823	592	Following 48 h outgrowth with probiotic
6m_24 h_GM	428,719	713	Control after 24 h outgrowth in GMM
6m_24 h_Pr	421,086	712	Following 24 h outgrowth with probiotic
6m_48 h_GM	251,829	661	Control after 48 h outgrowth in GMM
6m_48 h_Pr	326,559	711	Following 48 h outgrowth with probiotic
7m_24 h_GM	524,362	668	Control after 24 h outgrowth in GMM
7m_24 h_Pr	700,920	745	Following 24 h outgrowth with probiotic
12m_24 h_GM	535,503	452	Control after 24 h outgrowth in GMM
12m_24 h_Pr	611,090	490	Following 24 h outgrowth with probiotic
